# Agreement of keratometry measurements for toric IOL planning in routine clinical practice: KR-800, IOLMaster 500, and Pentacam HR

**DOI:** 10.1371/journal.pone.0356995

**Published:** 2026-08-24

**Authors:** Yeoeun Lim, Moonwon Hwang

**Affiliations:** 1 Cognitive Science Program, College of Arts and Sciences, Indiana University, Bloomington, Indiana, United States of America; 2 Department of Ophthalmology, Inje University Busan Paik Hospital, Inje University College of Medicine, Busan, Republic of Korea; Saarland University, GERMANY

## Abstract

Accurate keratometry is essential for toric intraocular lens (IOL) planning, yet most prior studies evaluated inter-device agreement under standardized research conditions that may not reflect everyday practice. This retrospective study evaluated keratometry agreement among the KR-800, IOLMaster 500, and Pentacam HR under real-world clinical conditions in 227 eyes of cataract surgery candidates. Corneal astigmatism was converted to power vector components: J0 (with/against-the-rule astigmatism) and J45 (oblique astigmatism). Population-level agreement was assessed using linear mixed-effects models and intraclass correlation coefficients (ICC), while individual-level agreement was evaluated using Bland-Altman analysis. Agreement in toric IOL candidacy and the predicted residual astigmatism arising from inter-device differences were also examined. No significant population-level differences were observed among devices (J0: *p* = 0.138; J45: *p* = 0.233), with ICC values of 0.894 for J0 and 0.748 for J45, respectively. However, the 95% limits of agreement were wide (0.80–1.21 D). When any device pair was considered, the predicted residual astigmatism exceeded 0.50 D in 64.8% of patients, and the Pentacam HR classified fewer eyes as toric IOL candidates than the reflection-based devices. Among toric IOL candidates (≥1.0 D astigmatism), 21–33% demonstrated axis differences exceeding 10°, a threshold associated with clinically relevant reduction in astigmatic correction. Axis differences were inversely correlated with astigmatism magnitude (Spearman’s rho = −0.220 to −0.272, all *p*-values < .001). The three devices showed acceptable agreement at the population level, and notably, the average magnitude of disagreement was limited. At the individual level, however, differences exceeded clinically relevant thresholds in a meaningful proportion of patients, including some toric IOL candidates. Using a single device consistently throughout surgical planning may be prudent, although the clinical impact of these differences was not directly assessed and warrants prospective validation.

## Introduction

Toric intraocular lenses (IOLs) have become essential tools for correcting corneal astigmatism during cataract surgery [1]. Preoperative corneal astigmatism of 1.5 diopters (D) or greater is present in approximately 20% of patients undergoing cataract surgery [2], highlighting the clinical importance of accurate astigmatism assessment. The effectiveness of toric IOLs depends on accurate preoperative keratometry, as errors in cylinder power or axis alignment can reduce the intended astigmatic correction. Notably, a misalignment of 10 degrees results in approximately a one-third reduction in cylindrical correction [3,4]. Accordingly, inter-device differences in measured astigmatism magnitude or axis may translate into different toric IOL choices and/or different expected residual astigmatism for individual patients, even when mean values are similar at the population level.

Several keratometry and optical biometry devices are currently available for preoperative assessment, yet they rely on different measurement principles. The Pentacam HR (OCULUS Optikgeräte GmbH, Wetzlar, Germany) employs rotating Scheimpflug imaging to capture the anterior segment, whereas the IOLMaster 500 (Carl Zeiss Meditec AG, Jena, Germany) uses partial coherence interferometry with six-point reflection-based keratometry. The KR-800 (Topcon Corporation, Tokyo, Japan) is an automated kerato-refractometer that measures corneal curvature using reflection-based technology similar to conventional keratometers [5,6]. Given these differences in acquisition and measurement geometry, device outputs may not be identical, particularly for axis estimation that is directly relevant to toric alignment.

Previous studies comparing keratometry devices have reported conflicting conclusions regarding their interchangeability. Some cautioned against interchangeable use because individual-level differences in cylinder power and axis can be substantial [6–8]. Others suggested that these devices may be interchangeable for toric IOL selection, reporting no clinically significant difference in IOL power calculation despite measurable differences in keratometry values [9,10]. Prior comparative studies, however, were typically performed under standardized research protocols with optimized measurement conditions. In routine clinical practice, measurements are often obtained by multiple technicians, in variable examination sequences, and without repeated averaging—conditions that may increase inter-device variability. Whether reported levels of agreement generalize to such real-world settings remains unclear.

The purpose of this study was to evaluate the agreement of anterior corneal astigmatism from keratometry among the KR-800, IOLMaster 500, and Pentacam HR in cataract surgery candidates, with particular emphasis on the clinical implications for toric IOL planning, including direct axis comparison. We employed power vector analysis [11] to enable proper statistical comparison of astigmatism data and applied clinically meaningful thresholds to assess the practical interchangeability of these devices. Measurements were obtained under real-world clinical conditions, rather than optimized research protocols, to provide applicable information for clinical decision-making in everyday surgical planning.

## Methods

### Study design and patients

This retrospective study was conducted at Inje University Busan Paik Hospital, Busan, Republic of Korea. Medical records of patients who underwent preoperative evaluation for cataract surgery between August 2021 and December 2023 were reviewed. Data were accessed for research purposes from 11 August 2025–30 September 2025. The corresponding author had access to information that could identify individual participants during data collection; however, all data were de-identified prior to analysis. The study protocol adhered to the tenets of the Declaration of Helsinki and was approved by the Institutional Review Board of Inje University Busan Paik Hospital (IRB No. 2024-12-035). The requirement for informed consent was waived due to the retrospective nature of the study.

Inclusion criteria were: (1) age ≥ 19 years, (2) scheduled cataract surgery, and (3) complete keratometry measurements from all three devices (KR-800, IOLMaster 500, and Pentacam HR) obtained within the same day. Exclusion criteria were: (1) history of corneal surgery including refractive surgery, (2) corneal pathology affecting keratometry measurements (e.g., corneal opacity, keratoconus, pterygium), (3) contact lens wear within 2 weeks before examination, and (4) poor-quality measurements, defined as an acquisition error on the KR-800, a low-reliability warning on the IOLMaster 500, or a Pentacam HR quality specification rating other than OK. When both eyes of a patient met the inclusion criteria, the eye that underwent surgery first was included to maintain statistical independence.

### Keratometry devices

Three keratometry devices were used in this study. The KR-800 (Topcon Corporation, Tokyo, Japan) is an automated kerato-refractometer that measures corneal curvature by analyzing the reflection of illuminated rings projected onto the cornea over an approximately 3.0 mm diameter zone. For each eye it automatically acquired at least three consecutive readings and displayed their internal average, and this averaged value was recorded. The IOLMaster 500 (Carl Zeiss Meditec AG, Jena, Germany) uses telecentric keratometry with image analysis of six reflected light spots arranged hexagonally over an approximately 2.5 mm diameter zone [5]. Keratometry was obtained in the standard keratometry mode with default settings, and the instrument averaged three consecutive readings and alerted the examiner when reliability was low or a reading could not be obtained. The signal-to-noise ratio threshold applied by this instrument to axial length acquisition does not apply to keratometry, and no such setting was altered. The Pentacam HR (OCULUS Optikgeräte GmbH, Wetzlar, Germany) is a rotating Scheimpflug camera that reconstructs the corneal surface from cross-sectional images of the anterior segment. Simulated keratometry (SimK) values for the anterior corneal surface were derived from the sagittal curvature map over the central 3.0 mm zone.

Measurements were performed by trained ophthalmic technicians during routine preoperative evaluation, and the examiner for a given device was not standardized. The KR-800 was performed first, after which the IOLMaster 500 and the Pentacam HR were performed in an order that varied with clinic workflow rather than a fixed sequence. The three measurements were obtained in a single session on the same day, without a scheduled rest interval between devices, and were typically completed within 30 minutes. One device output was recorded per instrument. Each recorded value therefore represents the instrument’s internal average of consecutive readings, and acquisitions were not repeated or averaged across separate sessions by the operator. Keratometry was routinely obtained at least one hour after instillation of a mydriatic agent. No topical anesthetic, fluorescein, or other ocular dye was applied before measurement, and artificial tears were not instilled for the examination. KR-800 readings recorded without an acquisition error were accepted, as this instrument does not provide a separate post-measurement quality index, and for the Pentacam HR only examinations with a quality specification rating of OK were included.

### Data collection and power vector conversion

Corneal astigmatism data, including cylinder power and axis, were extracted from each device. All three devices reported corneal astigmatism in minus-cylinder form, so the recorded cylinder power and axis were entered into the power vector conversion without any change of sign or axis convention. To enable valid statistical comparison of astigmatism measurements, the conventional cylinder notation (cylinder power and axis) was converted to power vector components (J0 and J45) using the method described by Thibos et al. [11]:


$$\begin{array}{c} J\text{0}=-\left(\frac{C}{\text{2}}\right)\times \;cos(\text{2}\alpha ) \end{array}$$ (1)

$$\begin{array}{c} J\text{45}=-\left(\frac{C}{\text{2}}\right)\times \;sin(\text{2}\alpha ) \end{array}$$ (2)

where C is the cylinder power (in minus-cylinder form) and α is the axis in degrees. The J0 component represents the Jackson cross-cylinder at axis 0° and 90° (with-the-rule/against-the-rule astigmatism), while J45 represents the Jackson cross-cylinder at axis 45° and 135° (oblique astigmatism). The corneal astigmatism magnitude was calculated as $\text{2}\times ({J\text{0}}^{\text{2}}+{J\text{4}\text{5}}^{\text{2}})$, and the average astigmatism magnitude across the three devices was used for subgroup stratification. The vector difference between device pairs was calculated as: $\left[(\Delta {J\text{0}}^{\text{2}})+{(\Delta J\text{4}\text{5}}^{\text{2}})\right]$.

### Axis difference calculation

To provide clinically intuitive information for toric IOL axis planning, direct axis differences between device pairs were also calculated. Due to the 180-degree periodicity of astigmatism axis, the axis difference was computed as: $\text{min}(\left|{Axis}_{\text{1}}-{Axis}_{\text{2}}\right|,\;\text{1}\text{8}\text{0}\circ \;-\left|{Axis}_{\text{1}}-{Axis}_{\text{2}}\right|)$.

This formula ensures that the calculated difference correctly accounts for the circular nature of axis measurements (e.g., the difference between 5° and 175° is calculated as 10°, not 170°). Axis differences were analyzed for both the entire study population and the subgroup of patients with average corneal astigmatism ≥1.0 D, as this threshold is commonly used to indicate candidacy for toric IOL implantation [12]. Clinical thresholds of ≤5°, > 10°, and >15° were applied based on previous literature suggesting that axis misalignment of 10° or more can result in clinically significant reduction of the intended astigmatic correction [3,4]. Differences between low (<1.0 D) and high (≥1.0 D) astigmatism groups were compared using Mann-Whitney U tests, as axis differences exhibited right-skewed distributions. The relationship between astigmatism magnitude and axis difference was assessed using Spearman correlation.

### Statistical analysis

All statistical analyses were performed using R version 4.3.1 (R Foundation for Statistical Computing, Vienna, Austria). All tests were two-tailed, and a *p*-value < 0.05 was considered statistically significant. The distribution of each variable was assessed using the Shapiro-Wilk test. Data are presented as mean ± standard deviation (SD) unless otherwise specified. Median with interquartile range (IQR) was used for skewed distributions. All 227 patients had complete measurements from all three devices; no missing data were present.

Population-level agreement among the three devices was evaluated using linear mixed-effects models (LMMs) with device as a fixed effect and patient as a random effect, accounting for the repeated-measures structure of the data. Models were fitted using restricted maximum likelihood (REML) estimation with the *lme4* package [13]; *p*-values and degrees of freedom were calculated using Satterthwaite’s approximation via the *lmerTest* package [14]. The intraclass correlation coefficient (ICC) was calculated as the ratio of between-patient variance to total variance (between-patient variance + within-patient variance) derived from the LMM variance components, with 95% confidence intervals obtained using Fisher’s z transformation. ICC values were interpreted according to Koo and Li [15]: values <0.50 indicate poor, 0.50–0.75 moderate, 0.75–0.90 good, and >0.90 excellent reliability, respectively. As pairwise comparisons were conducted only when the overall F-test was significant, no adjustment for multiple comparisons was applied.

Individual-level agreement between device pairs was assessed using Bland-Altman analysis with the parametric approach (mean difference ± 1.96 × SD), which is the standard method and remains robust for large samples [16]. The 95% confidence intervals for the limits of agreement were calculated using the approximate standard error method. The presence of proportional bias was evaluated by ordinary least squares regression of the differences against the means, with a statistically significant slope (*p* < 0.05) indicating magnitude-dependent disagreement. Systematic bias was assessed using one-sample t-tests.

Clinical significance was assessed using threshold analysis. For axis differences, the proportion of patients exceeding clinically relevant thresholds (≤5°, > 10°, > 15°) was calculated for both the overall population and the toric IOL candidate subgroup (≥1.0 D astigmatism). Threshold-exceedance proportions were reported with 95% Wilson confidence intervals.

To explore whether the observed inter-device differences could affect decisions in toric IOL planning, two analyses were performed post hoc. Agreement in toric IOL candidacy, defined per device as corneal astigmatism of at least 1.0 D, was assessed with Cohen’s and Fleiss’ kappa and the McNemar exact test. In addition, the predicted residual astigmatism that would arise if a toric IOL were planned on one device while another represented the true cornea was calculated as twice the power vector difference (2 × √[(ΔJ0)² + (ΔJ45)²]) and expressed in cylinder diopters; it therefore represents a re-expression of the vector difference analysis rather than an independent measure. These values were compared with thresholds of 0.50, 0.75, and 1.00 D, informed by the association between residual astigmatism and visual outcomes reported by Schallhorn et al. [17], and the association between predicted residual astigmatism and astigmatism magnitude was assessed with Spearman’s rank correlation.

Sensitivity analyses were performed using robust linear mixed models with Huber-type weighting (*robustlmm* package [18]) to verify that results were not unduly influenced by outliers. The maximum absolute difference between fixed effect estimates from standard and robust LMMs was calculated to assess the stability of the findings.

## Results

### Study population and measurement characteristics

A total of 227 eyes from 227 patients were included in the analysis. The characteristics of the study population are summarized in Table 1. The mean age was 68.1 ± 11.9 years (range: 19–90 years), and 52.0% were female (*n* = 118). The mean corneal astigmatism averaged across the three devices was 1.15 ± 0.77 D (median: 0.97 D; IQR: 0.63–1.43 D; range: 0.20–6.41 D). Among the 227 patients, 109 (48.0%) had corneal astigmatism ≥1.0 D, representing potential candidates for toric IOL implantation. The distributions of J0 and J45 components deviated from normality for all three devices (Shapiro-Wilk test, all *p*-values < 0.05).

**Table 1:** Demographic and Clinical Characteristics of the Study Population.

Characteristic	Value (*N* = 227)
Age (years)	
Mean ± SD	68.1 ± 11.9
Range	19–90
Sex, *n* (%)	
Female	118 (52.0)
Male	109 (48.0)
Corneal astigmatism (D), average across devices	
Mean ± SD	1.15 ± 0.77
Median (IQR)	0.97 (0.63–1.43)
Range	0.20–6.41
Astigmatism by device (D), mean ± SD	
KR-800	1.10 ± 0.73
IOLMaster 500	1.19 ± 0.82
Pentacam HR	1.03 ± 0.84
J0 by device (D), mean ± SD	
KR-800	0.02 ± 0.57
IOLMaster 500	−0.02 ± 0.64
Pentacam HR	0.01 ± 0.60
J45 by device (D), mean ± SD	
KR-800	0.00 ± 0.34
IOLMaster 500	−0.02 ± 0.34
Pentacam HR	0.00 ± 0.28
Toric IOL candidates (≥1.0 D), *n* (%)	109 (48.0)

Abbreviations: D: diopters; IQR: interquartile range; SD: standard deviation.

### Population-level agreement

Linear mixed-effects models revealed no statistically significant differences among the three devices for either J0 or J45 components. For J0, the overall F-test for device effect was not significant [*F* (2, 452) = 1.99, *p* = 0.138]. Similarly, for J45, the device effect was not significant [*F* (2, 452) = 1.46, *p* = 0.233]. As the overall tests were not significant, no pairwise comparisons were conducted.

The intraclass correlation coefficient for J0 was 0.894 (95% CI: 0.865–0.918), indicating good agreement according to the criteria proposed by Koo and Li [15], with 89.4% of the total variance attributable to between-patient differences rather than between-device differences. For J45, the ICC was 0.748 (95% CI: 0.685–0.800), indicating moderate agreement. Sensitivity analyses using robust linear mixed models yielded similar results; the maximum difference between standard and robust fixed effect estimates was 0.045 D, confirming that the findings were not driven by outliers.

### Individual-level agreement

**Fig 1:**
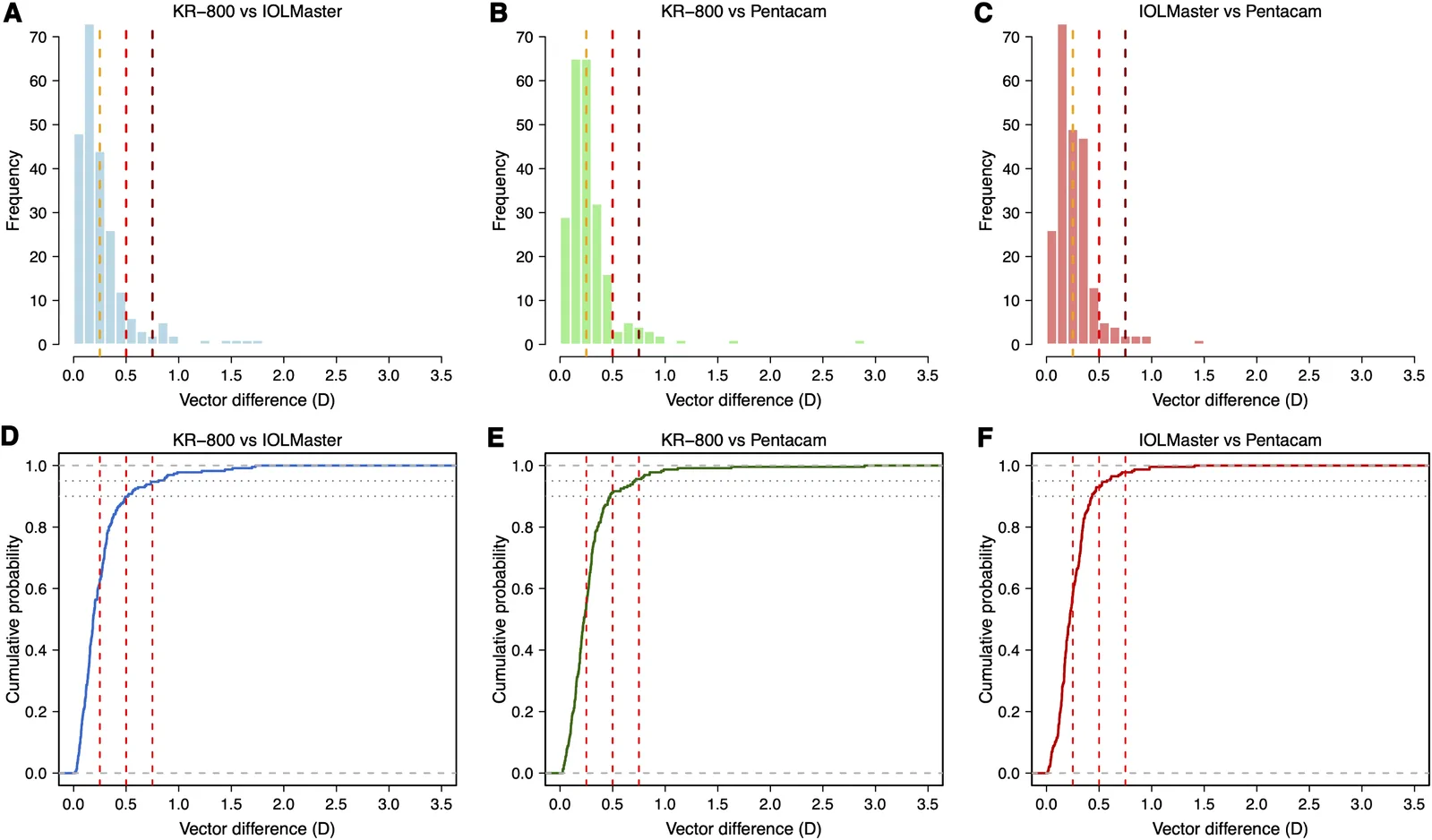
Bland-Altman Plots for J0 and J45 Power Vector Components. Plots show agreement between device pairs for J0 (A–C) and J45 (D–F) components: (A, D) KR-800 versus IOLMaster 500; (B, E) KR-800 versus Pentacam HR; (C, F) IOLMaster 500 versus Pentacam HR. The solid blue line represents the mean difference, and the dashed red lines represent the 95% limits of agreement (mean ± 1.96 × SD, LoA). The green line indicates the regression line for proportional bias assessment, shown only when statistically significant (*p* < 0.05). Each point represents one patient (*N* = 227).

Bland-Altman analysis revealed variability at the individual patient level despite the absence of significant population-level differences (Fig 1 and Table 2). The mean differences between device pairs were small, ranging from −0.031 D to 0.033 D for J0 and −0.022 D to 0.023 D for J45. However, the 95% limits of agreement (LoA) were wider, with widths ranging from 0.796 D to 1.214 D across all comparisons. For J0, the widest 95% LoA was observed between KR-800 and Pentacam HR (width: 1.214 D), followed by KR-800 and IOLMaster 500 (width: 1.117 D), and IOLMaster 500 and Pentacam HR (width: 0.920 D). For J45, IOLMaster 500 versus Pentacam HR showed the narrowest limits (width: 0.796 D).

**Table 2:** Bland-Altman Agreement Analysis for J0 and J45 Components.

Component	Device comparison	Mean diff (D)	SD (D)	95% LoA (D)	Width (D)	Slope	*P*-value
J0	KR-800 vs. IOLMaster 500	0.033	0.285	−0.526 (−0.590, −0.462) to 0.591 (0.527, 0.656)	1.117	−0.126	< 0.001
	KR-800 vs. Pentacam HR	0.002	0.310	−0.605 (−0.675, −0.536) to 0.609 (0.539, 0.678)	1.214	−0.060	0.101
	IOLMaster 500 vs. Pentacam HR	−0.031*	0.235	−0.491 (−0.544, −0.438) to 0.429 (0.376, 0.482)	0.920	0.067	0.009
J45	KR-800 vs. IOLMaster 500	0.023	0.241	−0.449 (−0.503, −0.395) to 0.494 (0.440, 0.548)	0.943	0.001	0.985
	KR-800 vs. Pentacam HR	0.001	0.237	−0.464 (−0.517, −0.411) to 0.465 (0.412, 0.519)	0.929	0.219	< 0.001
	IOLMaster 500 vs. Pentacam HR	−0.022	0.203	−0.420 (−0.466, −0.375) to 0.376 (0.330, 0.422)	0.796	0.209	< 0.001

Abbreviations: D: diopters; diff: difference; LoA: limits of agreement; SD: standard deviation.

Notes:Values in parentheses are the 95% CIs of the corresponding lower and upper limits of agreement, calculated using the approximate standard error method. *Systematic bias significant [*t*(226) = −2.00, *p* = 0.047, one-sample t-test]. Slope represents the regression coefficient from ordinary least squares regression of differences against means; significant slope (*p* < 0.05) indicates proportional bias.

Proportional bias was detected in four of six device pair comparisons. For J0, a significant negative slope was observed for KR-800 versus IOLMaster 500 (slope = −0.126, *p* < 0.001), and a significant positive slope was observed for IOLMaster 500 versus Pentacam HR (slope = 0.067, *p* = 0.009). KR-800 versus Pentacam HR did not show significant proportional bias (*p* = 0.101). For J45, significant proportional bias was detected for KR-800 versus Pentacam HR (slope = 0.219, *p* < 0.001) and IOLMaster 500 versus Pentacam HR (slope = 0.209, *p* < 0.001), but not for KR-800 versus IOLMaster 500 (*p* = 0.985).

Systematic bias (mean difference significantly different from zero) was detected only for the IOLMaster 500 versus Pentacam HR comparison in J0 [mean difference = −0.031 D, *t*(226) = −2.00, *p* = 0.047].

### Vector difference analysis

**Fig 2:**
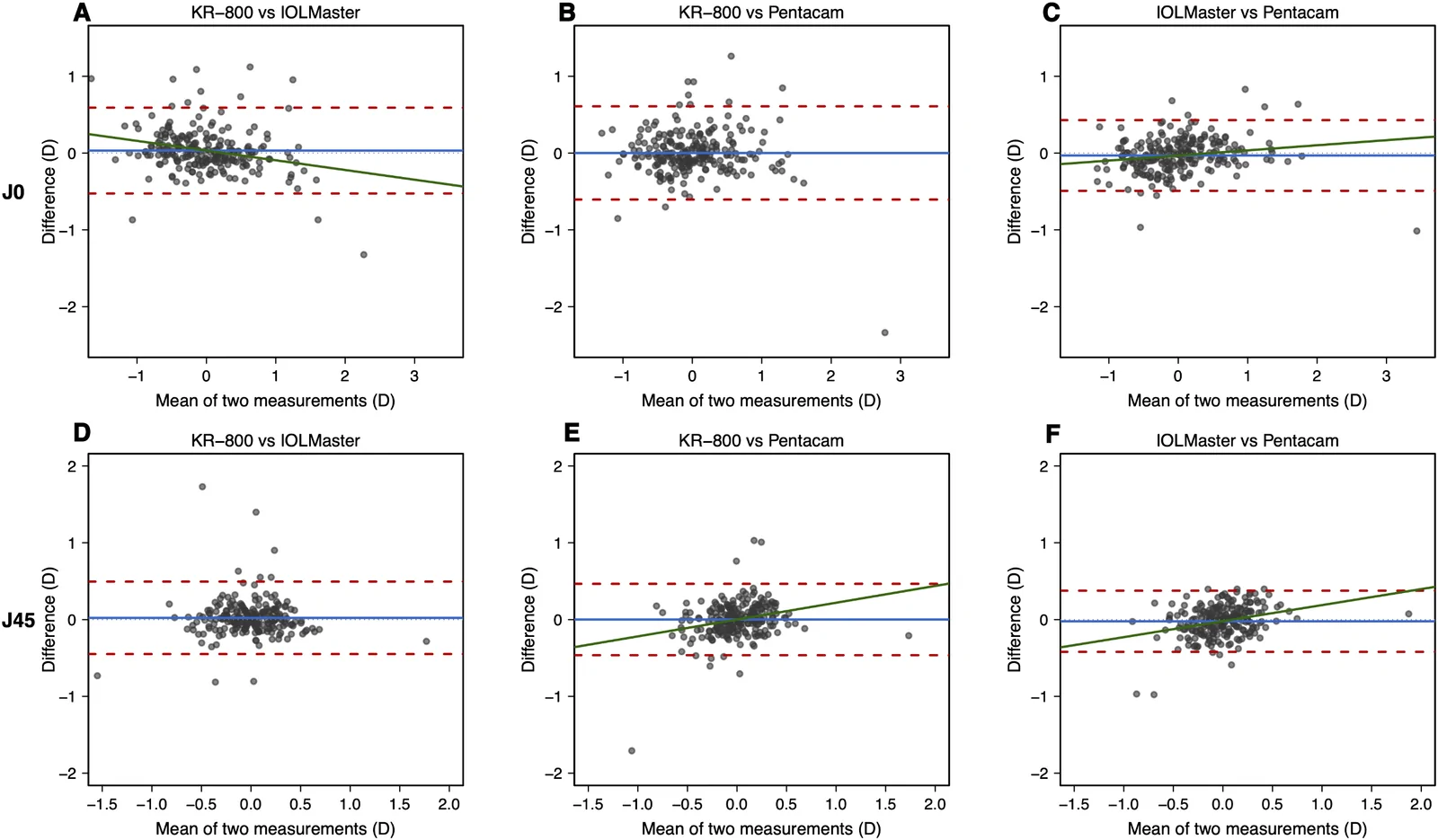
Vector Difference Distributions between Device Pairs. Histograms (A–C) and cumulative distribution functions (D–F) of vector differences: (A, D) KR-800 versus IOLMaster 500; (B, E) KR-800 versus Pentacam HR; (C, F) IOLMaster 500 versus Pentacam HR. Vector difference was calculated as $\left[(\Delta {J\text{0}}^{\text{2}})+{(\Delta J\text{4}\text{5}}^{\text{2}})\right]$. Vertical dashed lines mark power vector differences of 0.25 D (orange), 0.50 D (red), and 0.75 D (dark red) for reference. The corresponding predicted residual astigmatism in cylinder diopters is twice these power vector values. *N* = 227 for all comparisons.

The combined vector differences between device pairs showed positively skewed distributions (Fig 2). The median vector differences were 0.186 D (IQR: 0.116–0.311 D) for KR-800 versus IOLMaster 500, 0.228 D (IQR: 0.148–0.320 D) for KR-800 versus Pentacam HR, and 0.221 D (IQR: 0.146–0.327 D) for IOLMaster 500 versus Pentacam HR. These values are expressed in power vector (J0/J45) units. The corresponding residual astigmatism in cylinder diopters, together with its comparison to published clinical thresholds, is reported under Clinical relevance for toric IOL planning.

### Axis agreement analysis

**Fig 3:**
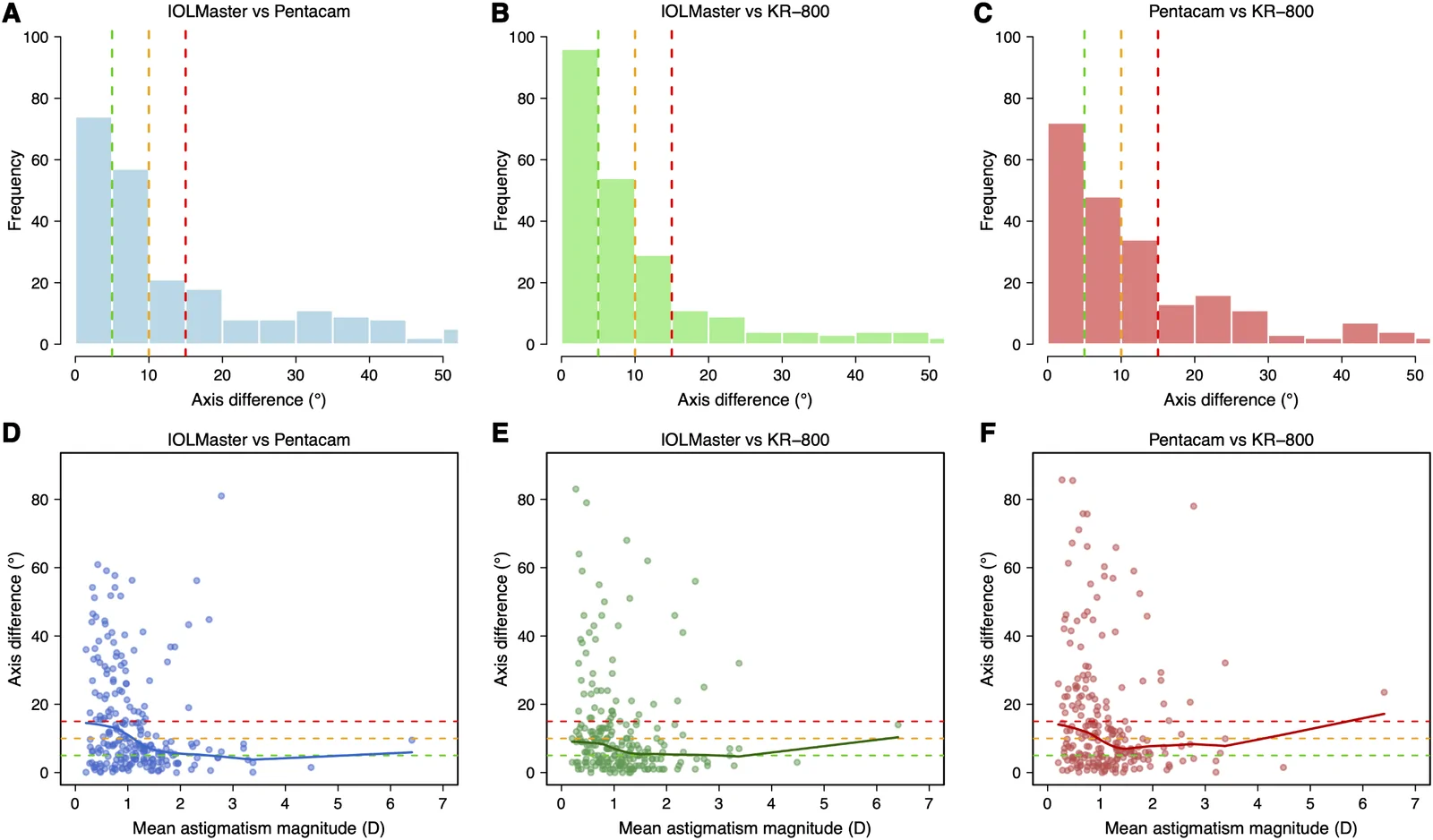
Axis Difference Analysis between Device Pairs. Distribution of axis differences (A–C) and relationship between astigmatism magnitude and axis difference (D–F): (A, D) IOLMaster 500 versus Pentacam HR; (B, E) IOLMaster 500 versus KR-800; (C, F) Pentacam HR versus KR-800. Vertical dashed lines in panels A–C and horizontal dashed lines in panels D–F indicate thresholds at 5° (green), 10° (orange), and 15° (red). LOWESS smoothing curves (solid lines) in panels D–F illustrate the non-linear relationship between astigmatism magnitude and axis difference. *N* = 227 for all comparisons.

Direct axis differences between device pairs are presented in Table 3 and Fig 3. For the entire study population (*N* = 227), the mean axis differences ranged from 12.1° to 15.9° depending on the device pair. The proportion of patients with axis differences exceeding 10° ranged from 33.9% (IOLMaster 500 versus KR-800) to 47.1% (Pentacam HR versus KR-800).

**Table 3:** Axis Differences between Device Pairs by Astigmatism Group.

Device comparison	Overall population (*N* = 227)	Toric IOL candidates (≥1.0 D, *n* = 109)	≤5° (%)	>10° (%)	>15° (%)
	Mean ± SD (°)	Mean ± SD (°)	Overall / ≥1.0 D	Overall / ≥1.0 D	Overall / ≥1.0 D
IOLMaster 500 vs. Pentacam HR	14.7 ± 15.4	10.3 ± 13.2	32.6 / 42.2	42.3 / 24.8	33.0 / 16.5
IOLMaster 500 vs. KR-800	12.1 ± 14.9	9.1 ± 13.0	42.3 / 56.9	33.9 / 21.1	21.1 / 12.8
Pentacam HR vs. KR-800	15.9 ± 18.2	12.2 ± 15.7	31.7 / 37.6	47.1 / 33.0	32.2 / 20.2

Abbreviations: D: diopters; SD: standard deviation.

Notes: Axis difference was calculated as $\text{min}(\left|{Axis}_{\text{1}}-{Axis}_{\text{2}}\right|,\;\text{1}\text{8}\text{0}\circ \;-\left|{Axis}_{\text{1}}-{Axis}_{\text{2}}\right|)$ to account for the 180° periodicity of astigmatism axis.

Axis differences were significantly associated with astigmatism magnitude. Patients with corneal astigmatism <1.0 D (*n* = 118) had larger median axis differences than those with astigmatism ≥1.0 D (*n* = 109): 14.3° (IQR: 5.7°–30.3°) versus 6.1° (IQR: 3.0°–10.0°) for IOLMaster 500 versus Pentacam HR (Mann-Whitney U test, *W* = 8546.0, *p* < 0.001), 10.0° (IQR: 4.0°–17.8°) versus 5.0° (IQR: 2.0°–9.0°) for IOLMaster 500 versus KR-800 (*W* = 8567.5, *p* < 0.001), and 13.6° (IQR: 4.7°–25.0°) versus 6.4° (IQR: 3.5°–12.6°) for Pentacam HR versus KR-800 (*W* = 8179.5, *p* < 0.001). Spearman correlation analysis confirmed significant negative correlations between astigmatism magnitude and axis difference for all device pairs (Spearman’s rho = −0.220 to −0.272, all *p*-values < 0.001).

Among patients with corneal astigmatism ≥1.0 D (toric IOL candidates, *n* = 109), axis agreement improved but differences remained. In this subgroup, the mean axis differences were 10.3° ± 13.2° for IOLMaster 500 versus Pentacam HR, 9.1° ± 13.0° for IOLMaster 500 versus KR-800, and 12.2° ± 15.7° for Pentacam HR versus KR-800. The proportion of patients with axis differences exceeding 10° ranged from 21.1% (IOLMaster 500 versus KR-800; 95% CI 14.5 to 29.7) to 33.0% (Pentacam HR versus KR-800; 95% CI 24.9 to 42.3). The proportion exceeding 15° ranged from 12.8% (95% CI 7.8 to 20.4) to 20.2% (95% CI 13.7 to 28.7) depending on the device pair.

### Clinical relevance for toric IOL planning

To explore whether the observed inter-device differences could affect decisions in toric IOL planning, agreement in toric IOL candidacy and the predicted residual astigmatism were examined post hoc.

When each device was used independently to classify eyes as toric IOL candidates (corneal astigmatism of at least 1.0 D on that device), the candidacy rate was 53.3% for the KR-800, 52.0% for the IOLMaster 500, and 44.5% for the Pentacam HR. Pairwise agreement ranged from moderate to substantial (Cohen’s kappa 0.580 to 0.693), and agreement across all three devices was substantial (Fleiss’ kappa 0.653, 95% CI 0.575 to 0.729). Classification was discordant in 15.4% to 21.1% of eyes (Table 4). Of the discordant eyes, 66% to 83% had average corneal astigmatism within 0.75 to 1.25 D. The discordance was symmetric between the KR-800 and the IOLMaster 500 (McNemar *p* = 0.736) but asymmetric for both comparisons involving the Pentacam HR (*p* = 0.006 for each), with the Pentacam HR classifying fewer eyes as candidates.

**Table 4:** Toric IOL Candidacy Concordance Between Devices (*N* = 227).

Device comparison	Discordant (%)	Cohen’s κ (95% CI)	McNemar *p*	Discordant with 0.75–1.25 D (%)
KR-800 vs. IOLMaster 500	15.4	0.691 (0.597, 0.785)	0.736	66
KR-800 vs. Pentacam HR	21.1	0.580 (0.476, 0.684)	0.006	71
IOLMaster 500 vs. Pentacam HR	15.4	0.693 (0.600, 0.785)	0.006	83

Abbreviations: CI, confidence interval; D, diopters; κ, kappa.

Notes: Candidacy was defined per device as corneal astigmatism of at least 1.0 D on that device. Fleiss’ κ across all three devices was 0.653 (95% CI 0.575 to 0.729). Marginal candidacy rates were 53.3% (KR-800), 52.0% (IOLMaster 500), and 44.5% (Pentacam HR). McNemar *p* values are from exact binomial tests on the discordant cells. The final column gives the percentage of discordant eyes whose average corneal astigmatism fell within 0.75 to 1.25 D.

Among toric IOL candidates, the median predicted residual astigmatism ranged from 0.38 to 0.48 D across device pairs (Table 5). It exceeded 0.50 D in 37.6% to 47.7% of this subgroup, 0.75 D in 12.8% to 21.1%, and 1.00 D in 10.1% to 11.0%. In the overall sample, the predicted residual astigmatism exceeded 0.50 D for at least one device pair in 64.8% of eyes (*n* = 147), 0.75 D in 28.6% (*n* = 65), and 1.00 D in 16.3% (*n* = 37). Predicted residual astigmatism showed a weak positive association with astigmatism magnitude (Spearman’s rho = 0.08 to 0.15, significant in two of three comparisons).

**Table 5:** Predicted Residual Astigmatism Between Devices in Toric IOL Candidates (≥1.0 D, *n* = 109), in Cylinder Diopters.

Device comparison	Median (IQR), D	> 0.50 D, % (95% CI)	> 0.75 D, % (95% CI)	> 1.00 D, % (95% CI)
KR-800 – IOLMaster 500	0.376 (0.235–0.652)	39.4 (30.8–48.8)	21.1 (14.5–29.7)	11.0 (6.4–18.3)
KR-800 – Pentacam HR	0.479 (0.313–0.671)	47.7 (38.6–57.0)	21.1 (14.5–29.7)	10.1 (5.7–17.2)
IOLMaster 500 – Pentacam HR	0.438 (0.299–0.644)	37.6 (29.1–47.0)	12.8 (7.8–20.4)	10.1 (5.7–17.2)

Abbreviations: CI, confidence interval; D, diopters; IQR, interquartile range.

Notes:Predicted residual astigmatism equals twice the power vector difference, expressed in cylinder diopters. Proportion confidence intervals are Wilson intervals.

## Discussion

This study evaluated the agreement of keratometry measurements among three commonly used devices, KR-800, IOLMaster 500, and Pentacam HR, under routine clinical conditions, with specific focus on the clinical implications for toric IOL planning. The three devices showed acceptable agreement at the population level, and the average magnitude of inter-device disagreement was limited. Because this agreement held under routine clinical conditions rather than an optimized research protocol, it suggests that the population-level agreement reported in controlled studies may also hold in everyday practice. At the individual level, however, the 95% limits of agreement were wide, with widths of approximately 0.8 to 1.2 D across the power vector components, and inter-device differences exceeded clinically relevant thresholds in a proportion of patients, including some toric IOL candidates in whom axis differences could affect alignment. These observations may have implications for toric IOL planning, although the present study did not directly assess whether the differences would alter the toric IOL selected for an individual patient or the postoperative refractive outcome.

Our findings regarding keratometry agreement are consistent with previous reports showing no significant mean differences between devices. For instance, Karunaratne [9] found no clinically significant difference in IOL power calculations between Pentacam and IOLMaster 500. Srivannaboon et al. [10] also reported a similar magnitude of astigmatism measurements between AL-Scan and IOLMaster 500, with no clinically significant difference in overall toric IOL selection. Similarly, Sayed and Alsamman [19] found good agreement in anterior chamber depth and keratometry readings between Pentacam and IOLMaster in the context of phakic IOL calculation. In the present study, the ICC values indicated good agreement for J0 (0.894) but only moderate agreement for J45 (0.748). The lower ICC for J45 may reflect the lower prevalence and smaller magnitude of oblique astigmatism in the study population, which could make precise measurement more susceptible to noise. The ICC, however, depends on the between-patient variance of the sample [15] and does not by itself establish that the devices are interchangeable, since a high ICC mainly indicates that differences between patients are large relative to differences between devices. The Bland-Altman limits of agreement and the threshold analyses are therefore more directly relevant to whether the devices can be used interchangeably in an individual patient [16].

Notably, the 95% LoA observed in our study appear wider than those reported in previous controlled studies using power vector analysis. Wang et al. [20] reported 95% LoA of ≤±0.31 D for both J0 and J45 across eight keratometry devices, including IOLMaster, Pentacam, and Topcon autokeratometer, in healthy young adults. Kiraly et al. [21] reported excellent repeatability (ICC 0.974–0.999) for keratometry measurements between IOLMaster 700, Pentacam HR, and iDesign; however, their use of IOLMaster 700 rather than the IOLMaster 500 employed in our study warrants caution in direct comparison. Our findings of 95% LoA widths ranging from 0.920 to 1.214 D for J0 and 0.796 to 0.943 D for J45 in a real-world cataract population appear wider than these prior reports. This difference may reflect additional sources of variability present in routine clinical practice, including multiple examiners, non-standardized examination sequences, a single recorded measurement per device rather than repeated operator-averaged acquisitions, and an older cataract population with potentially compromised ocular surfaces. These findings suggest that agreement data from controlled research settings may not fully capture the variability encountered in everyday practice.

The presence of proportional bias in four of six device comparisons indicates that inter-device disagreement may not be constant across the measurement range; disagreement appeared to vary systematically with astigmatism magnitude. For instance, the negative slope observed for KR-800 versus IOLMaster 500 in J0 suggests that the devices may agree more closely at lower J0 values and diverge more at higher values, potentially corresponding to greater discrepancy in patients with higher with-the-rule or against-the-rule astigmatism. These findings suggest that simple correction factors may not adequately address inter-device differences across all patients. Systematic bias was detected only for the IOLMaster 500 versus Pentacam HR comparison in J0, indicating that most device pairs showed no consistent directional difference. This suggests that the observed inter-device variability may be primarily random rather than systematic, which could make simple calibration adjustments unlikely to fully resolve the discrepancies.

The clinical significance of inter-device differences can be considered in relation to the residual astigmatism that they would introduce. According to Schallhorn et al. [17], in monofocal IOL patients, residual astigmatism of 0.25–0.50 D was associated with 1.7 times higher odds of not achieving 20/20 uncorrected visual acuity, and 0.75–1.00 D was associated with 6.1 times higher odds. When the predicted residual astigmatism arising from inter-device differences was expressed in corneal cylinder terms, it exceeded 0.50 D in 64.8% of patients, 0.75 D in 28.6%, and 1.00 D in 16.3% for at least one device pair. This metric, however, is a re-expression of the inter-device vector difference and assumes that the planned correction is otherwise perfect. It therefore reflects the astigmatism attributable to selecting one device rather than another and does not by itself demonstrate that a different toric IOL would have been chosen or that the postoperative refraction would differ. The magnitude of disagreement also varied by device pair. The KR-800 versus Pentacam HR comparison showed the widest 95% limits of agreement (width 1.214 D for J0), and the IOLMaster 500 versus Pentacam HR comparison the narrowest (0.920 D for J0 and 0.796 D for J45), indicating relatively closer agreement between the latter pair. Taken together, these findings suggest that interchanging devices during planning could, in some patients, introduce astigmatic differences in the range that Schallhorn et al. [17] associated with reduced uncorrected acuity, although the present study did not directly measure this clinical impact.

Inter-device differences also affected the dichotomous decision of whether a patient met the 1.0 D threshold commonly used to indicate toric IOL candidacy. Agreement on candidacy was moderate to substantial, yet 15.4% to 21.1% of patients were classified differently depending on the device pair. This discordance should be interpreted with caution, because the median corneal astigmatism in our sample (0.97 D) lay close to the 1.0 D cutoff, so most discordant eyes fell within a narrow band around the threshold (0.75 to 1.25 D) rather than reflecting broad disagreement between devices. A directional difference was nonetheless evident and was not explained by this boundary effect, as the Pentacam HR classified significantly fewer eyes as candidates than the two reflection-based devices (McNemar test, *p* = 0.006 for both comparisons), consistent with the lower mean astigmatism it recorded. These observations indicate that, for patients with borderline astigmatism, the device used can influence whether a toric IOL is considered, although whether this would change the final surgical decision was not assessed.

Inter-device axis differences were common in the overall population, with 33.9–47.1% of patients showing differences greater than 10° between device pairs. These proportions are broadly consistent with previous reports. Lee et al. [7] reported that 30% of eyes showed axis differences exceeding 10° between Pentacam and IOLMaster, and Srivannaboon et al. [10] found similar proportions (32.8–34.3%) between AL-Scan and IOLMaster 500. The overall figure is influenced by eyes with low astigmatism, in which the axis is more sensitive to measurement noise, so the comparison most relevant to toric IOL planning is the subgroup of candidates. Even in the subgroup with corneal astigmatism ≥1.0 D (toric IOL candidates), 21.1–33.0% still demonstrated axis differences exceeding 10°, and 12.8–20.2% exceeded 15°. These proportions may be clinically relevant, because a 10° misalignment of a toric IOL has been reported to reduce the intended cylindrical correction by approximately one-third [3,4].

Axis differences were inversely related to astigmatism magnitude. Patients with low astigmatism (<1.0 D) showed median axis differences approximately twice as large as those with higher astigmatism (≥1.0 D), a pattern consistent with Srivannaboon et al. [10], who reported axis differences of 15–20° in the low cylinder group (<1.0 D) versus 3–7° in the high cylinder group (≥1.0 D). This may be explained by the geometric principle that the direction of a short astigmatic vector is more susceptible to measurement noise than that of a long vector, so axis determination becomes less reliable as astigmatism decreases. The large axis differences observed at very low astigmatism are, however, of limited clinical concern for toric planning, because these eyes fall below the threshold for toric IOL implantation and would not be treated on the basis of their axis. The clinically relevant range is therefore the borderline candidate group (approximately 1.0 to 1.5 D), in which the astigmatism is large enough to warrant a toric IOL yet small enough that the axis remains relatively unstable.

Several features of routine clinical measurement may have contributed to the wider limits of agreement observed in our study. First, the devices measure corneal curvature at slightly different zones, with the KR-800 and Pentacam HR using approximately 3.0 mm zones and the IOLMaster 500 a 2.5 mm hexagonal pattern [5]. Second, patient positioning varies between devices, and subtle differences in head tilt could affect axis measurements; Park et al. [22] reported that head tilt can induce cyclotorsion of similar magnitude. Third, tear film dynamics may change throughout the examination sequence, particularly in an older cataract population such as ours, in which the ocular surface is more often compromised. Finally, unlike controlled studies where a single experienced examiner performs all measurements, measurements by multiple technicians may introduce additional operator-dependent variability. These factors were not directly measured, however, and their relative contributions to the observed variability remain to be established.

Based on our findings, several clinical considerations could be suggested. For toric IOL candidates, measurements should ideally be obtained from a single device throughout the planning process to minimize inter-device variability. When device switching is unavoidable, clinicians should be aware that approximately two-thirds of patients showed a predicted residual astigmatism exceeding 0.50 D in at least one device comparison. For patients with borderline toric IOL candidacy, confirming axis measurements with a second device or repeating the measurement may be advisable, given the inverse relationship between astigmatism magnitude and axis reliability. Among the three devices studied, IOLMaster 500 and Pentacam HR showed the best agreement; if measurements from different devices must be compared, this combination may introduce relatively less variability.

This study has several limitations that should be acknowledged. The retrospective design and single-measurement protocol do not allow assessment of intra-device repeatability or direct comparison with optimized measurement conditions; however, this approach reflects actual clinical workflows and may enhance the generalizability of our findings. The tear film was also not optimized before measurement, and keratometry was performed after pupil dilation, as is routine in this setting. Because the three devices were measured sequentially within a single session, tear film changes between the measurements may have contributed to the observed inter-device variability, although this was not directly assessed. Additionally, all three devices assess anterior corneal curvature, and for the Pentacam HR we used simulated keratometry rather than total corneal power. The analysis was therefore limited to agreement in anterior keratometry and does not address posterior corneal astigmatism or total corneal power, which are increasingly incorporated into toric IOL planning. Future studies incorporating total corneal power measurements may provide further insights into inter-device agreement. Finally, the absence of postoperative refractive outcomes limits direct clinical validation of the observed discrepancies. Prospective studies correlating preoperative measurement differences with postoperative refractive results would help establish evidence-based tolerance thresholds for inter-device variability. Despite these limitations, the use of power vector analysis with clinically meaningful thresholds, combined with the large sample size, provides relevant insights for toric IOL planning in routine clinical settings.

In conclusion, the KR-800, IOLMaster 500, and Pentacam HR showed acceptable agreement at the population level, and the average magnitude of inter-device disagreement was limited even under the routine, non-experimental conditions of this study. At the individual level, however, the limits of agreement were wider than those reported in controlled research settings, and inter-device differences exceeded clinically relevant thresholds in a meaningful proportion of patients, including some toric IOL candidates. These findings suggest that the devices may not be interchangeable for toric IOL planning in individual patients, and that using a single device consistently throughout the planning process may be prudent. Standardized measurement protocols in routine clinical settings may also help reduce inter-device variability. Because the present study did not directly assess whether these differences altered toric IOL selection or postoperative refractive outcomes, the clinical implications remain inferential and warrant prospective validation.
