# Supplementary material for: Agreement of keratometry measurements for toric IOL planning in routine clinical practice: KR-800, IOLMaster 500, and Pentacam HR
Source: PLoS One. 2026 Aug 24;21(8):e0356995. doi: 10.1371/journal.pone.0356995 (PMC13502659; doi:10.1371/journal.pone.0356995)
Supplement: Supplementary file 1 [file pone.0356995.s002.pdf]

Response to Reviewers

We thank the Academic Editor and both Reviewers for their careful and constructive evaluation of our manuscript. We are encouraged that the Editor considers the work to have merit and that both Reviewers responded positively to the study's design and clinical relevance. We are particularly grateful for the thoughtful guidance on interpreting our findings more cautiously and on strengthening the manuscript in the context of toric IOL planning. We have revised the manuscript carefully in response to every point raised, and we detail the changes below. Page numbers refer to the revised manuscript with track changes.

1. Comments from the Editor

1.1. For the Manuscript

Please carefully read the comments raised by reviewer 1 regarding the over interpretation in the discussion and some ideas to improve the manuscript in the context of toric IOL planning.

Response: We are grateful to the Editor for highlighting these two priorities. We have taken Reviewer 1's concern about over-interpretation seriously and have revised the Discussion and Conclusion throughout, so that explanations which were not directly tested in this study are now presented as hypotheses rather than as demonstrated mechanisms, and so that the clinical implications are explicitly framed as inferential rather than directly established. In response to the suggestion to strengthen the manuscript in the context of toric IOL planning, we have added two analyses that the present data can validly support without relying on unmeasured assumptions. One examines inter-device concordance in toric IOL candidacy, namely whether each device would classify a given eye as a toric candidate at the commonly used 1.0 D threshold. The other quantifies the predicted residual astigmatism attributable to inter-device disagreement, expressed in corneal cylinder diopters and referenced to established clinical thresholds. We describe these revisions in detail in our point-by-point responses to Reviewer 1 below, where the Editor's two concerns are addressed most directly. We are grateful for guidance that has, in our view, meaningfully improved both the rigor and the clinical relevance of the manuscript.

1.2. For the Journal Requirements

Response:

1) PLOS ONE style requirements: We have carefully reviewed the PLOS ONE style templates and have revised our manuscript to ensure it meets all formatting requirements, including file naming conventions.
2) Citing recommended works: We have reviewed the Reviewers' comments for any specific publications recommended for citation. The Reviewers did not request the addition of particular references; their comments concerned how the findings should be interpreted and reported rather than which prior works should be cited. We have

nonetheless ensured that all claims in the revised manuscript remain appropriately supported by the cited literature.

3) ORCID iD: The ORCID iD of all authors has been verified in the submission system. Thank you for checking in this detail.
4) Financial disclosure: We confirm that there are no changes to our financial disclosure, and the original statement remains accurate.
5) Figures: We have reviewed the PLOS ONE figure guidelines and prepared all figures in accordance with the technical requirements.
6) Protocols.io: As this was a retrospective study based on routinely acquired clinical measurements rather than a prospective experimental protocol, no separate laboratory protocol was applicable, and we have therefore not deposited a protocol in protocols.io.

2. Comments from Reviewer 1

This manuscript addresses a clinically relevant question by evaluating the agreement of anterior corneal astigmatism measurements obtained with the KR-800, IOLMaster 500, and Pentacam HR in routine cataract surgery work-up. The study has several notable strengths, including a reasonably large sample size, the use of power-vector analysis for astigmatism comparison, and a meaningful distinction between population-level agreement and individual-level agreement. The finding that mean differences between devices are small while individual-level discrepancies may still be clinically relevant is important and potentially valuable for toric IOL planning. Overall, I consider the manuscript promising and potentially suitable for publication after revision. However, several issues should be addressed before acceptance. My main concern is that the discussion sometimes interprets the findings more strongly than the data directly support.

Response: We are sincerely grateful to Reviewer 1 for this thorough and encouraging assessment, and for recognizing the study as clinically relevant and soundly designed while offering guidance that has clearly strengthened it. We found these comments tremendously helpful, particularly in directing us toward more cautious interpretation and clearer methodological reporting, and we have revised the manuscript accordingly. We address each point in turn below.

2.1. Interpretation of the wider limits of agreement

In particular, the authors attribute the relatively wide limits of agreement to 'real-world' factors such as multiple technicians, variable examination sequence, lack of repeated averaging, older patient age, and compromised ocular surface conditions. These explanations are certainly plausible and clinically sensible, but they were not directly measured or tested in the present study. For that reason, this interpretation should be presented more cautiously and explicitly framed as a hypothesis rather than as a demonstrated explanation.

Response: We fully agree with the Reviewer, and we are grateful for this important point. These factors were neither measured nor manipulated in our retrospective design, and we agree that they should not have been presented as established explanations for the wider limits of agreement. We have revised the paragraph that discusses these conditions so that they are now framed explicitly as possible contributors rather than demonstrated causes. The paragraph now states that several features of routine clinical measurement "may have contributed to the wider limits of agreement observed in our study," and it closes with the explicit acknowledgment that "These factors were not directly measured, however, and their relative contributions to the observed variability remain to be established" (p.27). In the earlier paragraph that compares our limits of agreement with those of prior controlled studies, the same conditions are likewise presented only as a possible explanation, with the wider limits described as something that "may reflect" the additional variability present in routine clinical practice rather than as a demonstrated consequence of it (p.23). We hope these revisions present our interpretation as the hypothesis it is, in keeping with the Reviewer's guidance.

2.2. Clinical significance of the observed differences and additional analyses for toric IOL planning

A second important issue is that the clinical significance of the observed differences is inferred only indirectly. The manuscript uses vector-difference thresholds and prior literature on residual postoperative astigmatism to argue that the inter-device differences may be clinically meaningful for toric IOL planning. However, the study did not assess whether the measurement differences actually changed toric IOL cylinder selection, axis choice, predicted residual astigmatism, or postoperative refractive outcomes. Therefore, the conclusions regarding clinical impact should be moderated. Ideally, the manuscript would be strengthened by an additional analysis showing how often these inter-device differences would have resulted in a different toric IOL recommendation. If such an analysis is not feasible, the discussion and conclusion should make clearer that the clinical implications remain inferential rather than directly demonstrated.

Response: We are grateful to the Reviewer for this comment, which we regard as the most important of the review. We fully agree that, in the original manuscript, the clinical significance of the inter-device differences was inferred only indirectly, and that we did not assess whether the differences actually changed toric IOL cylinder selection, axis choice, predicted residual astigmatism, or postoperative refractive outcomes. We have responded in two complementary ways. Following the Reviewer's suggestion, we added analyses that bring the question of a different toric IOL recommendation closer to the data, to the extent that the present dataset can support such an analysis without relying on unmeasured assumptions. At the same time, we moderated the clinical-impact claims throughout the manuscript so that they are framed explicitly as inferential rather than directly demonstrated.

For the first, we added an analysis of inter-device concordance in toric IOL candidacy, reported in a new Results subsection ("Clinical relevance for toric IOL planning") and an accompanying table (pp.19–22). Because the decision of whether to implant a toric IOL is commonly made at a corneal astigmatism threshold of approximately 1.0 D, we classified each eye as a candidate or a non-candidate under each device and examined whether this dichotomous decision agreed across devices. Agreement was moderate to substantial (Cohen's kappa 0.580 to 0.693 across the three device pairs; Fleiss' kappa 0.653), yet 15.4% to 21.1% of eyes were classified differently depending on the device used. This speaks to the Reviewer's question, since a change in candidacy status represents a change in whether a toric IOL would be considered at all.

We were careful not to over-interpret this discordance. Because the median corneal astigmatism in our sample (0.97 D) lay very close to the 1.0 D cutoff, many eyes were near the threshold, and most discordant eyes fell within a narrow band around it, with approximately 66% to 83% of discordant eyes lying between 0.75 and 1.25 D. We report this explicitly so that the discordance is not misread as broad disagreement about candidacy. At the same time, a genuine directional difference was present that the boundary effect does not explain. The Pentacam HR classified significantly fewer eyes as candidates than the two reflection-based devices (McNemar test, $p = 0.006$ for both comparisons), consistent with the lower mean astigmatism it recorded. We therefore present these results as indicating that, for patients with borderline astigmatism, the device used can influence whether a toric IOL is considered, while stating explicitly that whether this would change the final surgical decision was not assessed.

We also added an analysis of the predicted residual astigmatism attributable to inter-device disagreement (p.20). If a toric correction is assumed to neutralize the astigmatism measured by one device, the residual astigmatism that would remain when a second device represents the true cornea is twice the power-vector difference between the two devices, expressed in corneal cylinder diopters. Computed across device pairs, this predicted residual astigmatism exceeded 0.50 D in 64.8% of eyes, 0.75 D in 28.6%, and 1.00 D in 16.3% in at least one comparison. We adopted this formulation because it allows the inter-device disagreement to be compared directly, and in consistent units, with the residual-astigmatism thresholds reported by Schallhorn et al., which the manuscript already referenced. In doing so we also expressed these threshold comparisons consistently in corneal cylinder diopters, since the original submission had compared power-vector differences with diopter thresholds without the factor-of-two conversion to corneal cylinder. We have been explicit, however, that this quantity is a re-expression of the measured inter-device difference under an idealized assumption of perfect correction, and that it does not by itself demonstrate that a different toric IOL would have been selected for any individual patient; we state this caveat in the Discussion (pp.24–25).

In the interest of transparency, we also wish to explain why we did not extend these analyses to specific toric IOL cylinder-grade selection or to postoperative outcomes. Assigning a specific cylinder grade to an eye requires information that was neither measured nor recorded in this study and that cannot be derived from corneal keratometry alone. It depends on the surgeon-specific surgically induced astigmatism, on the incision meridian, on the conversion from the corneal plane to the IOL plane (which is governed by the effective lens position), and on the cylinder steps of a particular IOL platform, which differ between manufacturers. Modern toric planning also increasingly incorporates posterior corneal astigmatism and total corneal power, which our anterior keratometric measurements do not capture. A grade-selection analysis would therefore reflect these assumed parameters as much as the device measurements themselves, and could constitute precisely the kind of over-interpretation that the Reviewer has appropriately cautioned against. We judged the dichotomous candidacy decision, which does not depend on platform or surgeon-specific parameters, to be the level at which our data can validly inform the question of a different recommendation. Postoperative refractive outcomes were likewise unavailable, because this was a retrospective study of preoperative measurement agreement, and we have identified the prospective correlation of preoperative inter-device differences with postoperative outcomes as the appropriate next step.

Finally, in keeping with the Reviewer's guidance, we moderated the clinical-impact claims throughout so that they remain inferential. The Abstract now notes that, although individual-level differences exceeded clinically relevant thresholds in a meaningful proportion of patients, including some toric candidates, the clinical impact of these differences was not directly assessed and warrants prospective validation (pp.2–3). The opening of the Discussion now states that the study did not directly assess whether the differences would alter the toric IOL selected or the postoperative refractive outcome (p.22). The Conclusion similarly states that, because the present study did not directly assess whether these differences altered toric IOL selection or postoperative refractive outcomes, the clinical implications remain inferential and warrant prospective validation (p.29). We are sincerely grateful to the Reviewer for guidance that has made the manuscript both more informative for toric IOL planning and more careful in its claims.

2.3. Emphasis on the toric IOL candidate subgroup in the axis analysis

The axis analysis is also somewhat difficult to interpret in the full cohort, because axis values are inherently less stable at low astigmatism magnitudes. The authors themselves demonstrate a significant inverse relationship between astigmatism magnitude and axis difference, which is an important and expected finding. However, from a clinical perspective, the subgroup with higher astigmatism is much more relevant for toric IOL planning than the entire study population. I would therefore recommend placing greater emphasis, both in the abstract and in the discussion, on the subgroup with corneal astigmatism of at least 1.0 D. In addition, the manuscript should explain more explicitly that large axis differences in eyes with very low astigmatism do not necessarily translate into clinically meaningful disagreement for toric planning.

Response: We are grateful to the Reviewer for this helpful guidance, with which we fully agree. The axis does become less stable as astigmatism decreases, and the significant inverse relationship between astigmatism magnitude and axis difference that we observed is itself an expression of this. For toric IOL planning, the subgroup with higher astigmatism is the relevant population, and we have revised both the Abstract (pp.2–3) and the Discussion (p.26) accordingly.

In the Abstract (p.2), the axis findings are presented for the toric IOL candidate subgroup, with 21% to 33% of candidates (corneal astigmatism of at least 1.0 D) showing axis differences exceeding 10°, so that the figures highlighted are the clinically relevant ones rather than those of the full cohort. In the Discussion, we have made the rationale for this emphasis explicit by adding to the axis paragraph that "The overall figure is influenced by eyes with low astigmatism, in which the axis is more sensitive to measurement noise, so the comparison most relevant to toric IOL planning is the subgroup of candidates" (p.26). The subgroup proportions, namely 21.1% to 33.0% exceeding 10° and 12.8% to 20.2% exceeding 15°, are retained as the clinically relevant figures.

We have also addressed the Reviewer's request to explain more explicitly why large axis differences in eyes with very low astigmatism do not necessarily imply clinically meaningful disagreement. In the paragraph on the inverse relationship, we have added that "The large axis differences observed at very low astigmatism are, however, of limited clinical concern for toric planning, because these eyes fall below the threshold for toric IOL implantation and would not be treated on the basis of their axis. The clinically relevant range is therefore the borderline candidate group (approximately 1.0 to 1.5 D), in which the astigmatism is large enough to warrant a toric IOL yet small enough that the axis remains relatively unstable" (p.27). We hope this clarifies that the large axis differences seen at very low magnitudes, while statistically real, are unlikely to affect toric IOL planning, and that attention is appropriately directed to the borderline candidate range, where clinical relevance and residual axis instability coincide.

2.4. Methodological clarifications

The authors should specify more clearly whether all cylinder values were converted into a uniform minus-cylinder notation before transformation into J0 and J45. Although this may be implied, it should be stated explicitly. It would also be helpful to describe in slightly greater detail exactly which keratometric outputs from each device were used, whether measurements were obtained before any drops or other interventions, and whether any additional quality-control procedures were applied beyond the device-specific quality flags. These details would improve transparency and reproducibility.

Response: We sincerely thank the Reviewer for these suggestions, which have helped us improve the transparency and reproducibility of the Methods. We have revised the relevant sections to address each point.

We have now stated explicitly that all three devices recorded corneal astigmatism in minus-cylinder notation, and that these values were used directly in the conversion to the J0 and J45 power-vector components. Because the outputs were already in a uniform minus-cylinder form, no transposition between plus- and minus-cylinder conventions was required before the transformation.

We have also described the keratometric output of each device in greater detail. For the KR-800, the value used was the reading produced automatically after at least three internal measurements. For the IOLMaster 500, we used the keratometry obtained in the standard measurement mode. For the Pentacam HR, the simulated keratometry was derived from the anterior sagittal curvature map at the 3.0 mm zone, and the Belin/Ambrósio enhanced ectasia display was not used. We have additionally clarified that a single recorded value per device was used, where "single" denotes one recorded measurement rather than repeated operator-averaged acquisitions, since the KR-800 and IOLMaster 500 values themselves represent internal device averages.

We have specified the conditions under which the measurements were obtained as well. Keratometry was routinely performed at least one hour after instillation of the mydriatic agent, and no topical anesthetic, dye, or artificial tears were applied before measurement. We have also noted in the Limitations that, given the retrospective design, the tear film was not optimized before measurement and keratometry was performed after pupil dilation, as is routine in this setting. We have added that, because the three devices were measured sequentially within a single session, tear film changes between the measurements may have contributed to the observed variability, although this was not directly assessed (p.28).

Finally, we have described the device-specific quality-control procedures more concretely. Only error-free readings from the KR-800 were used; for the IOLMaster 500, measurements flagged as unreliable by the instrument were excluded, and we note that the signal-to-noise ratio criterion of this device applies to axial-length acquisition rather than to keratometry; and for the Pentacam HR, only examinations with a quality specification rated as "OK" were included. Eyes with poor-quality measurements on any device were excluded, as stated in the exclusion criteria. We hope these additions provide the transparency and reproducibility that the Reviewer sought.

2.5. Statistical reporting: limits-of-agreement confidence intervals and the role of the ICC

The Methods state that 95% confidence intervals for the limits of agreement were calculated, but these intervals are not presented in Table 2. The authors should either report them or remove that statement. In addition, the role of the intraclass correlation coefficient should be explained more carefully. ICC can be useful as a descriptive measure, but it is not itself a direct measure of interchangeability and may be strongly influenced by between-subject variability. Since the manuscript already uses Bland-Altman analysis appropriately, it would help to clarify that the Bland-Altman results are more directly relevant to the clinical question of interchangeability.

Response: We are grateful to the Reviewer for these two points, both of which we have addressed. For the limits of agreement, we chose to report the 95% confidence intervals rather than remove the statement, since these intervals convey the precision of the limits and are clinically informative. The confidence intervals for both the lower and upper limits of agreement are now presented for every device-pair comparison in Table 2, so that the Methods and the table are consistent. For the intraclass correlation coefficient, we fully agree with the Reviewer's characterization and have revised the Discussion accordingly. We now explain that the ICC is a descriptive measure of relative reliability that depends on the between-patient variance of the sample, and that a high ICC does not by itself establish that two devices are interchangeable for an individual patient. The revised passage states that the ICC "depends on the between-patient variance of the sample and does not by itself establish that the devices are interchangeable," and that "the Bland-Altman limits of agreement and the threshold analyses are therefore more directly relevant" to the clinical question of interchangeability (p.23). In this way the manuscript now presents the ICC as a useful descriptive index while directing the clinical interpretation toward the Bland-Altman and threshold results, as the Reviewer recommended.

2.6. Citation in the Figure 2 legend

I also noted a likely citation error in the Figure 2 legend. The statement about the 0.50 D threshold being associated with reduced uncorrected visual acuity appears to cite reference 15, which is the Koo and Li paper on ICC interpretation, whereas this statement seems to correspond instead to reference 17. This should be corrected.

Response: We thank the Reviewer for catching this error. The Reviewer is correct that the statement about the 0.50 D threshold and reduced uncorrected visual acuity was attributed to reference 15 (Koo and Li), whereas the appropriate source is reference 17 (Schallhorn et al.). In revising the manuscript, we have removed this sentence from the Figure 2 legend, because the comparison of inter-device differences with clinically relevant thresholds has been reframed as predicted residual astigmatism in corneal cylinder diopters and relocated to the Results and Discussion, where it is now correctly attributed to Schallhorn et al. The mis-citation therefore no longer appears, and the Figure 2 legend now describes only the content of the figure.

2.7. Language and typographical editing

In addition, the manuscript would benefit from a careful language edit for minor stylistic and typographical improvements.

Response: We thank the Reviewer for this suggestion. We have carefully reviewed the entire manuscript and corrected minor stylistic and typographical issues to improve clarity and readability throughout, paying particular attention to consistency of terminology and notation. We hope the revised manuscript now reads clearly.

2.8. Scope of the study: anterior keratometric agreement

Finally, because the Pentacam data are based on simulated keratometry from the anterior corneal surface only, it would be useful to emphasize again that the study concerns agreement of anterior keratometric measurements rather than comprehensive modern toric planning approaches that may incorporate posterior corneal astigmatism.

Response: We agree with the Reviewer, and we are grateful for this clarifying point. We have revised the Limitations to make the anterior scope of the study explicit. The revised passage notes that all three devices assess anterior corneal curvature and that, for the Pentacam HR, we used simulated keratometry rather than total corneal power, so that the analysis is limited to agreement in anterior keratometry and "does not address posterior corneal astigmatism or total corneal power, which are increasingly incorporated into toric IOL planning" (pp.28–29). In this way the scope of our findings, namely agreement of anterior keratometric measurements rather than comprehensive posterior-inclusive toric planning, is now stated explicitly.

We deeply thank Reviewer 1 once more for this careful and constructive review. We agree that the revisions called for were primarily a matter of clearer methodological reporting and more cautious interpretation, and we believe that addressing them has substantially strengthened the manuscript.

3. Comments from Reviewer 2

This paper reports the agreement among machines in measuring astigmatism. AS it can be expected, the measured astigmatism varies across devices, and especially so because of the non-experimental conditions: only one measurement per machine, ocular surface not controlled, perfect centration not addressed.

Response: We are deeply grateful to our Reviewer, Dr. Roberto Bellucci, for this thoughtful assessment and for identifying what we agree is the principal contribution of the study.

While the outcome can be anticipated out of the existing literature, the value of the paper is in the limited variability detected in the examined patients. I would like the authors to add a comment about that.

Response: We thank the Reviewer for this valuable observation, with which we fully agree. The contribution of the study lies not in the expected finding that measurements differ across devices, but in the magnitude of that disagreement under routine, non-experimental conditions, which proved to be limited at the population level and in its average magnitude. We have added this point explicitly in the Abstract (pp.2–3), the opening of the Discussion (p.22), and the Conclusion (p.29), each of which now describes the average magnitude of inter-device disagreement as limited and the population-level agreement as acceptable.

At the same time, and as Reviewer 1 also emphasized, we have preserved the complementary observation that, although the average disagreement was limited, individual-level differences exceeded clinically relevant thresholds in a meaningful proportion of patients, including some toric IOL candidates. We hope this balanced framing reflects the Reviewer's point that the limited variability is itself the informative result, while remaining accurate about the individual-level differences that are relevant to toric IOL planning.

We would also note that the non-experimental conditions the Reviewer mentions, including the use of a single recorded measurement per device and the absence of standardized ocular surface preparation, are now described in the Methods (p.8) and considered, as possible contributors to the observed variability, in the Discussion (pp.27–28) and Limitations (pp.28–29).

Once again, we sincerely thank the Academic Editor and both Reviewers for their thoughtful and constructive comments, which have substantially improved the manuscript.
