# Supplementary material for: Agreement of keratometry measurements for toric IOL planning in routine clinical practice: KR-800, IOLMaster 500, and Pentacam HR
Source: PLoS One. 2026 Aug 24;21(8):e0356995. doi: 10.1371/journal.pone.0356995 (PMC13502659; doi:10.1371/journal.pone.0356995)
Supplement: Peer review history

# Peer review: Decision Letter 0

**DOI:** 10.1371/journal.pone.0356995.r001

**Authors:** Timo Eppig

25 May 2026

Dear Dr. Hwang,

Please submit your revised manuscript by Jul 09 2026 11:59PM. If you will need more time than this to complete your revisions, please reply to this message or contact the journal office at plosone@plos.org. When you're ready to submit your revision, log on to https://www.editorialmanager.com/pone/ and select the 'Submissions Needing Revision' folder to locate your manuscript file.

- A letter that responds to each point raised by the academic editor and reviewer(s). You should upload this letter as a separate file labeled 'Response to Reviewers'.
- A marked-up copy of your manuscript that highlights changes made to the original version. You should upload this as a separate file labeled 'Revised Manuscript with Track Changes'.
- An unmarked version of your revised paper without tracked changes. You should upload this as a separate file labeled 'Manuscript'.

If you would like to make changes to your financial disclosure, please include your updated statement in your cover letter. Guidelines for resubmitting your figure files are available below the reviewer comments at the end of this letter.

If applicable, we recommend that you deposit your laboratory protocols in protocols.io to enhance the reproducibility of your results. Protocols.io assigns your protocol its own identifier (DOI) so that it can be cited independently in the future. For instructions see: https://journals.plos.org/plosone/s/submission-guidelines#loc-laboratory-protocols. Additionally, PLOS ONE offers an option for publishing peer-reviewed Lab Protocol articles, which describe protocols hosted on protocols.io. Read more information on sharing protocols at https://plos.org/protocols?utm_medium=editorial-email&utm_source=authorletters&utm_campaign=protocols.

As the corresponding author, your ORCID iD is verified in the submission system and will appear in the published article. PLOS supports the use of ORCID, and we encourage all coauthors to register for an ORCID iD and use it as well. Please encourage your coauthors to verify their ORCID iD within the submission system before final acceptance, as unverified ORCID iDs will not appear in the published article. *Only*  the individual author can complete the verification step; PLOS staff *cannot*  verify ORCID iDs on behalf of authors.

We look forward to receiving your revised manuscript.

Kind regards,

Timo Eppig

Academic Editor

PLOS One

Journal Requirements:

When submitting your revision, we need you to address these additional requirements.

1. Please ensure that your manuscript meets PLOS ONE's style requirements, including those for file naming. The PLOS ONE style templates can be found at https://journals.plos.org/plosone/s/file?id=wjVg/PLOSOne_formatting_sample_main_body.pdf and https://journals.plos.org/plosone/s/file?id=ba62/PLOSOne_formatting_sample_title_authors_affiliations.pdf

If the reviewer comments include a recommendation to cite specific previously published works, please review and evaluate these publications to determine whether they are relevant and should be cited. There is no requirement to cite these works unless the editor has indicated otherwise.

[Note: HTML markup is below. Please do not edit.]

Reviewers' comments:

Reviewer's Responses to Questions

**Comments to the Author**

1. Is the manuscript technically sound, and do the data support the conclusions?

Reviewer #1: Yes

Reviewer #2: Yes

**********

2. Has the statistical analysis been performed appropriately and rigorously?

Reviewer #1: Yes

Reviewer #2: Yes

**********

3. Have the authors made all data underlying the findings in their manuscript fully available?

The PLOS Data policy

Reviewer #1: Yes

Reviewer #2: Yes

**********

4. Is the manuscript presented in an intelligible fashion and written in standard English?

Reviewer #1: Yes

Reviewer #2: Yes

**********

Reviewer #1: This manuscript addresses a clinically relevant question by evaluating the agreement of anterior corneal astigmatism measurements obtained with the KR-800, IOLMaster 500, and Pentacam HR in routine cataract surgery work-up. The study has several notable strengths, including a reasonably large sample size, the use of power-vector analysis for astigmatism comparison, and a meaningful distinction between population-level agreement and individual-level agreement. The finding that mean differences between devices are small while individual-level discrepancies may still be clinically relevant is important and potentially valuable for toric IOL planning. Overall, I consider the manuscript promising and potentially suitable for publication after revision. However, several issues should be addressed before acceptance. My main concern is that the discussion sometimes interprets the findings more strongly than the data directly support. In particular, the authors attribute the relatively wide limits of agreement to “real-world” factors such as multiple technicians, variable examination sequence, lack of repeated averaging, older patient age, and compromised ocular surface conditions. These explanations are certainly plausible and clinically sensible, but they were not directly measured or tested in the present study. For that reason, this interpretation should be presented more cautiously and explicitly framed as a hypothesis rather than as a demonstrated explanation. A second important issue is that the clinical significance of the observed differences is inferred only indirectly. The manuscript uses vector-difference thresholds and prior literature on residual postoperative astigmatism to argue that the inter-device differences may be clinically meaningful for toric IOL planning. However, the study did not assess whether the measurement differences actually changed toric IOL cylinder selection, axis choice, predicted residual astigmatism, or postoperative refractive outcomes. Therefore, the conclusions regarding clinical impact should be moderated. Ideally, the manuscript would be strengthened by an additional analysis showing how often these inter-device differences would have resulted in a different toric IOL recommendation. If such an analysis is not feasible, the discussion and conclusion should make clearer that the clinical implications remain inferential rather than directly demonstrated. The axis analysis is also somewhat difficult to interpret in the full cohort, because axis values are inherently less stable at low astigmatism magnitudes. The authors themselves demonstrate a significant inverse relationship between astigmatism magnitude and axis difference, which is an important and expected finding. However, from a clinical perspective, the subgroup with higher astigmatism is much more relevant for toric IOL planning than the entire study population. I would therefore recommend placing greater emphasis, both in the abstract and in the discussion, on the subgroup with corneal astigmatism of at least 1.0 D. In addition, the manuscript should explain more explicitly that large axis differences in eyes with very low astigmatism do not necessarily translate into clinically meaningful disagreement for toric planning. Several methodological points would also benefit from clarification. The authors should specify more clearly whether all cylinder values were converted into a uniform minus-cylinder notation before transformation into J0 and J45. Although this may be implied, it should be stated explicitly. It would also be helpful to describe in slightly greater detail exactly which keratometric outputs from each device were used, whether measurements were obtained before any drops or other interventions, and whether any additional quality-control procedures were applied beyond the device-specific quality flags. These details would improve transparency and reproducibility. There are also some issues in the statistical reporting. The Methods state that 95% confidence intervals for the limits of agreement were calculated, but these intervals are not presented in Table 2. The authors should either report them or remove that statement. In addition, the role of the intraclass correlation coefficient should be explained more carefully. ICC can be useful as a descriptive measure, but it is not itself a direct measure of interchangeability and may be strongly influenced by between-subject variability. Since the manuscript already uses Bland-Altman analysis appropriately, it would help to clarify that the Bland-Altman results are more directly relevant to the clinical question of interchangeability. I also noted a likely citation error in the Figure 2 legend. The statement about the 0.50 D threshold being associated with reduced uncorrected visual acuity appears to cite reference 15, which is the Koo and Li paper on ICC interpretation, whereas this statement seems to correspond instead to reference 17. This should be corrected. In addition, the manuscript would benefit from a careful language edit for minor stylistic and typographical improvements. Finally, because the Pentacam data are based on simulated keratometry from the anterior corneal surface only, it would be useful to emphasize again that the study concerns agreement of anterior keratometric measurements rather than comprehensive modern toric planning approaches that may incorporate posterior corneal astigmatism. In summary, this is a useful and clinically relevant study with an overall sound design and potentially publishable results. The major revisions required are less about fundamental flaws in the analysis and more about clearer methodological reporting and more cautious interpretation of the clinical implications. With these revisions, the manuscript would be substantially strengthened.

Reviewer #2: This paper reports the agreement among machines in measuring astigmatism. AS it can be expected, the measured astigmatism varies across devices, and especially so because of the non-experimental conditions: only one measurement per machine, ocular surface not controlled, perfect centration not addressed. While the outcome can be anticipated out of the existing literature, the value of the paper is in the limited variability detected in the examined patients. I would like the authors to add a comment about that.

**********

what does this mean?). If published, this will include your full peer review and any attached files.

If you choose “no”, your identity will remain anonymous but your review may still be made public.

**Do you want your identity to be public for this peer review?**  For information about this choice, including consent withdrawal, please see our Privacy Policy

Reviewer #1: No

Reviewer #2: **Yes:**  Roberto Bellucci

**********

[NOTE: If reviewer comments were submitted as an attachment file, they will be attached to this email and accessible via the submission site. Please log into your account, locate the manuscript record, and check for the action link "View Attachments". If this link does not appear, there are no attachment files.]

To ensure your figures meet our technical requirements, please review our figure guidelines: https://journals.plos.org/plosone/s/figures

You may also use PLOS’s free figure tool, NAAS, to help you prepare publication quality figures: https://journals.plos.org/plosone/s/figures#loc-tools-for-figure-preparation.

NAAS will assess whether your figures meet our technical requirements by comparing each figure against our figure specifications.

# Author comment: Author response to Decision Letter 1

**DOI:** 10.1371/journal.pone.0356995.r002

10 Jun 2026

We sincerely thank the Academic Editor, Dr. Timo Eppig, and both Reviewers for their thoughtful and constructive comments. We have revised the manuscript carefully in response to every point raised. A detailed, point-by-point response is provided in the accompanying Response to Reviewers file, and all changes are marked in the tracked-changes version of the revised manuscript.

Attachment Submitted filename: Response to Reviewers.pdf

# Peer review: Decision Letter 1

**DOI:** 10.1371/journal.pone.0356995.r003

**Authors:** Timo Eppig

12 Aug 2026

Agreement of keratometry measurements for toric IOL planning in routine clinical practice: KR-800, IOLMaster 500, and Pentacam HR

PONE-D-26-04357R1

Dear Dr. Hwang,

We’re pleased to inform you that your manuscript has been judged scientifically suitable for publication and will be formally accepted for publication once it meets all outstanding technical requirements.

Within one week, you’ll receive an e-mail detailing the required amendments. When these have been addressed, you’ll receive a formal acceptance letter and your manuscript will be scheduled for publication.

An invoice will be generated when your article is formally accepted. Please note, if your institution has a publishing partnership with PLOS and your article meets the relevant criteria, all or part of your publication costs will be covered. Please make sure your user information is up-to-date by logging into Editorial Manager at Editorial Manager® and clicking the ‘Update My Information' link at the top of the page. For questions related to billing, please contact billing support.

If your institution or institutions have a press office, please notify them about your upcoming paper to help maximize its impact. If they’ll be preparing press materials, please inform our press team as soon as possible -- no later than 48 hours after receiving the formal acceptance. Your manuscript will remain under strict press embargo until 2 pm Eastern Time on the date of publication. For more information, please contact onepress@plos.org.

Kind regards,

Timo Eppig

Academic Editor

PLOS One

Additional Editor Comments (optional):

Reviewers' comments:

Reviewer's Responses to Questions

**Comments to the Author**

Reviewer #1: All comments have been addressed

Reviewer #2: All comments have been addressed

**********

2. Is the manuscript technically sound, and do the data support the conclusions?

Reviewer #1: Yes

Reviewer #2: Yes

**********

3. Has the statistical analysis been performed appropriately and rigorously?

Reviewer #1: I Don't Know

Reviewer #2: Yes

**********

4. Have the authors made all data underlying the findings in their manuscript fully available?

The PLOS Data policy

Reviewer #1: Yes

Reviewer #2: Yes

**********

5. Is the manuscript presented in an intelligible fashion and written in standard English?

Reviewer #1: Yes

Reviewer #2: Yes

**********

Reviewer #1: Tahnk you for the invitation to review the revised version of themanuscript. No further comments. All comments have been adressed.

Reviewer #2: The paper has been modified and the analysis extended. Now it can be read both in a simple way only to pick up suggestions or in a more precise way taking into account the figures.

**********

what does this mean?). If published, this will include your full peer review and any attached files.

If you choose “no”, your identity will remain anonymous but your review may still be made public.

**Do you want your identity to be public for this peer review?**  For information about this choice, including consent withdrawal, please see our Privacy Policy

Reviewer #1: No

Reviewer #2: No

**********

# Editor report: Acceptance letter

**DOI:** 10.1371/journal.pone.0356995.r004

**Authors:** Timo Eppig

PONE-D-26-04357R1

PLOS One

Dear Dr. Hwang,

I'm pleased to inform you that your manuscript has been deemed suitable for publication in PLOS One. Congratulations! Your manuscript is now being handed over to our production team.

At this stage, our production department will prepare your paper for publication. This includes ensuring the following:

* All references, tables, and figures are properly cited

* All relevant supporting information is included in the manuscript submission,

* There are no issues that prevent the paper from being properly typeset

You will receive further instructions from the production team, including instructions on how to review your proof when it is ready. Please keep in mind that we are working through a large volume of accepted articles, so please give us a few days to review your paper and let you know the next and final steps.

Lastly, if your institution or institutions have a press office, please let them know about your upcoming paper now to help maximize its impact. If they'll be preparing press materials, please inform our press team within the next 48 hours. Your manuscript will remain under strict press embargo until 2 pm Eastern Time on the date of publication. For more information, please contact onepress@plos.org.

You will receive an invoice from PLOS for your publication fee after your manuscript has reached the completed accept phase. If you receive an email requesting payment before acceptance or for any other service, this may be a phishing scheme. Learn how to identify phishing emails and protect your accounts at https://explore.plos.org/phishing.

If we can help with anything else, please email us at customercare@plos.org.

Thank you for submitting your work to PLOS One and supporting open access.

Kind regards,

PLOS ONE Editorial Office Staff

on behalf of

Prof. Dr. Timo Eppig

Academic Editor

PLOS One
